# Dysregulated A to I RNA editing and non-coding RNAs in neurodegeneration

**DOI:** 10.3389/fgene.2012.00326

**Published:** 2013-01-22

**Authors:** Minati Singh

**Affiliations:** Department of Internal Medicine, University of IowaIowa City, IA, USA

**Keywords:** RNA editing, ADARs, non-coding RNAs, microRNAs, snoRNAs, long non-coding RNA

## Abstract

RNA editing is an alteration in the primary nucleotide sequences resulting from a chemical change in the base. RNA editing is observed in eukaryotic mRNA, transfer RNA, ribosomal RNA, and non-coding RNAs (ncRNA). The most common RNA editing in the mammalian central nervous system is a base modification, where the adenosine residue is base-modified to inosine (A to I). Studies from ADAR (adenosine deaminase that act on RNA) mutants in *Caenorhabditis elegans*, *Drosophila*, and mice clearly show that the RNA editing process is an absolute requirement for nervous system homeostasis and normal physiology of the animal. Understanding the mechanisms of editing and findings of edited substrates has provided a better knowledge of the phenotype due to defective and hyperactive RNA editing. A to I RNA editing is catalyzed by a family of enzymes knows as ADARs. ADARs modify duplex RNAs and editing of duplex RNAs formed by ncRNAs can impact RNA functions, leading to an altered regulatory gene network. Such altered functions by A to I editing is observed in mRNAs, microRNAs (miRNA) but other editing of small and long ncRNAs (lncRNAs) has yet to be identified. Thus, ncRNA and RNA editing may provide key links between neural development, nervous system function, and neurological diseases. This review includes a summary of seminal findings regarding the impact of ncRNAs on biological and pathological processes, which may be further modified by RNA editing. NcRNAs are non-translated RNAs classified by size and function. Known ncRNAs like miRNAs, smallRNAs (smRNAs), PIWI-interacting RNAs (piRNAs), and lncRNAs play important roles in splicing, DNA methylation, imprinting, and RNA interference. Of note, miRNAs are involved in development and function of the nervous system that is heavily dependent on both RNA editing and the intricate spatiotemporal expression of ncRNAs. This review focuses on the impact of dysregulated A to I editing and ncRNAs in neurodegeneration.

## INTRODUCTION

Environmental signals provoke changes in gene expression in a mechanism that includes epigenetic-mediated gene regulation (Kubota et al., 2012; Miyake et al., 2012). Epigenetic regulation of gene expression has key roles in development, stress responses, and plasticity of the central nervous system (CNS). Epigenetic modifications include RNA editing and chromatin remodeling (i.e., histone modifications), and DNA methylation (Zhou et al., 2008; Luco and Misteli, 2011; Luco et al., 2011). Increasing evidence demonstrates that long non-coding RNAs (lncRNAs) are directed to the sites of action in the genome, suggesting that lncRNAs may be involved in regulation of methylation and chromatic remodeling (Luco and Misteli, 2011; Luco et al., 2011). In the CNS, dysregulation of these critical epigenetic processes leads to the pathogenesis of a broad range of neurological and psychiatric diseases (Mattick and Gagen, 2001; Clark, 2007).

**FIGURE 1:**
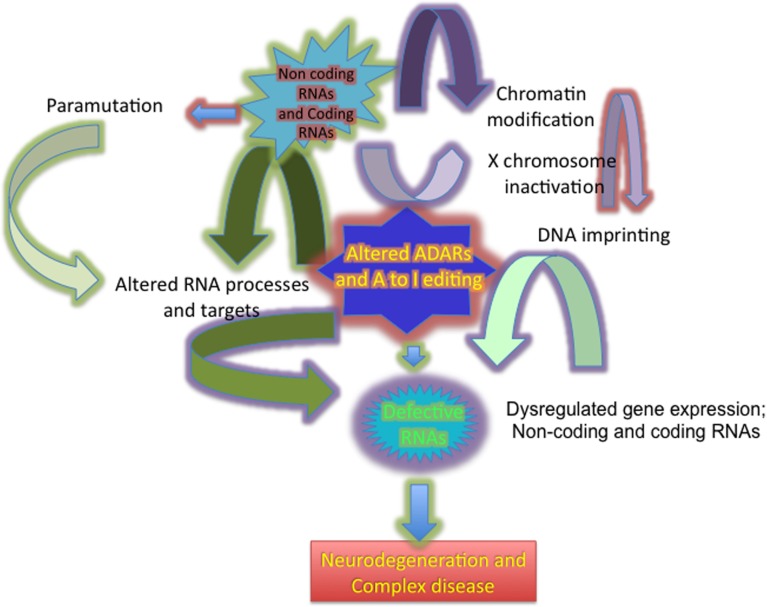
**Diagram showing the broad impact of dysregulated ADARs in altering A to I editing, producing defective RNAs and altering gene expression in neurodegeneration**.

Numerous classes of regulatory non-coding RNA (ncRNA) molecules contribute to the intricate biological system organization and gene regulatory networks that collectively allow normal functioning of the CNS. Dysregulation of these complex gene regulatory networks plays a significant role in the pathogenesis of common neurodegenerative disease such as Alzheimer’s disease (AD), Parkinson disease (PD), Huntington disease (HD), and amyotrophic lateral sclerosis (ALS; Akbarian et al., 1995b; Hideyama et al., 2012). In eukaryotes, a unifying theme in all of these genetic diseases is alterations in RNA regulation at multiple levels, including transcriptional changes, RNA editing (Burns et al., 1997; Emeson and Singh, 2001; Bass, 2002; Blow et al., 2006; Beal et al., 2007; Barraud and Allain, 2012), post-transcriptional gene silencing (Mette et al., 2000; Pal-Bhadra et al., 2002; Matzke et al., 2007; Carthew and Sontheimer, 2009; Ghildiyal and Zamore, 2009), X chromosome dosage compensation (Yang and Kuroda, 2007; Kanduri et al., 2009; Fedoriw et al., 2012), germ cell reprogramming (Migicovsky and Kovalchuk, 2011; Guibert et al., 2012), and para-mutation (Chandler, 2007; Cuzin et al., 2008; **Figure 1**).

Non-coding RNAs either receive or transmit information (Salta and De Strooper, 2012), which is often achieved by duplex structures formed by base pairing with complementary sequences of RNA and DNA; these duplexes can be in the form of either RNA:RNA or RNA:DNA complexes (Mattick, 2003; Mattick and Makunin, 2005). RNA-induced silencing complexes (RISC) or RNA editing by ADAR (adenosine deaminase that act on RNA) enzymes recognizes these duplex structures and process them by different mechanisms (Simpson and Emeson, 1996; Bass, 2002; Heale et al., 2009). Intersection of RNA editing in the RNA interference process is observed in both plants and animals (Luciano et al., 2004; Blow et al., 2006; Nishikura, 2006; Heale et al., 2009). Editing is induced by a variety of small ncRNAs such as endogenous small interfering RNAs (siRNAs), PIWI-interacting RNAs (piRNAs), and short transcripts that sit adjacent to promoter (i.e., promoter-associated RNAs; Han et al., 2007) and transcription initiation RNAs (Mehler, 2008). Furthermore, targeted RNA editing in the 3′ untranslated region (UTR) can affect stability, translation, or localization of mRNAs (Kuersten and Goodwin, 2003; Sie and Kuchka, 2011; Irimia et al., 2012). Taken together, these findings support the hypothesis that there is cross talk between RNA editing and the silencing machinery (Scadden and Smith, 2001; Blow et al., 2006; Nishikura, 2006; Ohman, 2007; Heale et al., 2009).

## DYSREGULATED A–I EDITING IN NEURODEGENERATION

Adenosine (A) to inosine (I) conversion in mRNA transcripts (A to I RNA editing) is catalyzed by a family of enzymes known as ADARs. Since inosine has the same base pairing properties as guanosine (G), the transcription and translational machinery recognizes I as a G. Hence, a silent mutation is created at the level of mRNA. In general, A to I editing most frequently targets repetitive RNA sequences located within the introns and 5′ and 3′ UTR to alter both sequence and structure of RNA. A to I editing is widespread and essential for normal life and development (Valente and Nishikura, 2005; Horsch et al., 2011). The ADAR gene family and A-to-I RNA editing deregulation, which results in uncorrected forms of hyper- or hypoediting, has been implicated in a spectrum of neurodevelopmental, neurodegenerative, and neuropsychiatric disorders, strongly suggesting diverse roles in post-transcriptional gene regulation (Valente and Nishikura, 2005).

A to I RNA editing of targeted substrates in the nervous system can alter functional properties of proteins, silence constitutive activity, and modulate RNA translation, localization, and stability (Paul and Bass, 1998; Bass, 2002). In addition to changing codons in mRNA, A to I editing also has the capacity to modulate splicing sites, small nucleolar RNA (sno-RNA) precursors, endogenous antisense RNAs, microRNA (miRNA) target diversity, miRNA and ncRNA processing, and ribonucleoprotein complex targets (Rueter et al., 1999; Bass, 2002; Levanon et al., 2004; Blow et al., 2006; Kawahara et al., 2006; Kishore and Stamm, 2006; Yang et al., 2006; Heale et al., 2009). ADAR involvement in chromatin modification and possibly in genomic imprinting and X-chromosome inactivation has been proposed, indicating that there is interplay between these processes and RNA-based silencing mechanisms (Fernandez et al., 2005; Valente and Nishikura, 2005). However, when edited mRNA is translated, editing alters biological properties and functions of proteins, such as ligand-gated receptors α -amino-3-hydroxy-5-methyl-4-isoxazolepropionic acid (AMPA) receptor (GluR-B subunit), serotonin 2C receptor, GABA_A_ receptor (alpha 3 subunit), and the potassium voltage-gated ion channel KV1.1. It is of note that, they all play an important role in nerve cell function (Emeson and Singh, 2001; Bass, 2002). ADAR2 activity can be modulated by sequestration in the nucleolus and nucleolus-nucleoplasm shuffling (Desterro et al., 2003; Sansam et al., 2003) and ADAR mRNA itself is subject to self-editing, known as auto-editing, which restricts its function in the adult *Drosophila* and mice (Rueter et al., 1999; Keegan et al., 2005; Feng et al., 2006).

In mammals ADARs are differentially expressed during organogenesis (Paupard et al., 2000; Jacobs et al., 2009). ADAR3 is restricted to the brain, whereas ADAR2 and ADAR1 are ubiquitously expressed but preferentially expressed in the CNS (Bass, 2002). During progressive stages of nervous system maturation, RNA editing also displays complex and dynamic profiles of sub-cellular localization and spatiotemporal expression (Bernard et al., 1999; Paupard et al., 2000; Sansam et al., 2003; Jacobs et al., 2009). Furthermore, both the behavioral state and genetic background can modify RNA editing (Englander et al., 2005). Changing environmental signals including inflammation and feedback regulation also modifies the activity and molecular profiles of ADARs (Bass, 2002; Yang et al., 2003; Valente and Nishikura, 2005). The potential central roles of RNA editing in brain evolution as well as gene–environmental interactions during nervous system neural maturation comes from studies showing environmentally responsive forms of ADARs. Specifically, the p150 long cytoplasmic isoform of ADAR1, which selectively targets endogenous antisense RNA pathways, is interferon-inducible, and ADAR3 exhibits selective regional, developmental, and mature nervous system expression. Taken together, these data identify unique modulatory roles and substrate specificity for ADARs (Melcher et al., 1996; Chen et al., 2000). ADAR2 has been found to contain inositol hexaphosphate (IP_6_) buried within its enzyme core (Macbeth et al., 2005). Moreover, amino acids that coordinate IP_6_ in ADAR2 are also conserved in adenosine deaminases that act on transfer RNAs (ADATs), and IP_6_ is required for ADAT activity (Macbeth et al., 2005), thereby linking ADAR2 to cell signaling pathways.

Multiple isoforms of ADAR1 and ADAR2 exists in the cell (Bass, 2002). ADAR1 displays preferential tissue-specific promoter utilization, whereas ADAR2 exists as multiple spliced isoforms generated by alternative splicing, which results in expression of a broad array of protein species with unique enzymatic properties and remarkable molecular diversity (George et al., 2005; Kawahara et al., 2005). ADARs are normally found as functional dimers that can either homo or hetero dimerize with their own isoforms (Chilibeck et al., 2006; Poulsen et al., 2006; Valente and Nishikura, 2007). However, dimerization of ADAR3 requires additional CNS environmental cues (Cho et al., 2003). Different ADARs can also edit multiple different sites on the same RNA species, resulting in diverse functional outcomes (Valente and Nishikura, 2005). Studies with transgenic mouse embryos that are deficient in both ADAR and ADARB1 activity revealed that deficiency of ADARB1 leads to accumulation of specific miRNAs and corresponding targets, thereby suggesting an important role for ADARs in miRNA biogenesis (Luciano et al., 2004; Yang et al., 2006; Ohman, 2007; Heale et al., 2009; Alon et al., 2012; Vesely et al., 2012). Furthermore ADARs binding alone can affect miRNA biogenesis and function and RNA interference in the nervous system (Heale et al., 2009; Paro et al., 2012). The biological roles of ADAR3 are particularly interesting due to its broad substrate specificity (binding single-stranded as well as double-stranded RNA) and localization that is restricted to brain regions and post-mitotic neurons (Melcher et al., 1996; Chen et al., 2000). ADAR3 can act as a dominant negative regulator for both ADAR1 and ADAR2 activity *in vitro,* thereby suggesting that correct expression levels of ADARs are required for optimal editing. Furthermore, ADAR3 can form heterodimers with both ADAR1 and ADAR2, providing mechanistic insight into the functional complexity associated with these enzymes in the brain (Chen et al., 2000).

A to I editing occurs more frequently in human transcripts than that was previously hypothesized. The majority of editing is now known to be in the Alu repeats, which are found in introns, intergenic transcripts, and UTRs and are believed to form duplex structures with ncRNAs (Athanasiadis et al., 2004; Kim et al., 2004b; Levanon et al., 2004; Blow et al., 2006). The most abundantly edited human transcripts are predominantly confined to the primate-specific Alu repeats, which are found in thousands of genes (Neeman et al., 2006; Chen and Carmichael, 2008; Chen et al., 2008). Interestingly, the editing levels in mouse and humans differ due in part to a higher divergence of mouse small interspersed nuclear elements (SINE) repeats and primate-specific Alu repeats (Alon et al., 2012; Vesely et al., 2012). However, two recent studies that estimated the total level of editing in the mouse have found that this is not the case (Kim et al., 2004a; Eisenberg et al., 2005). It is important to note that other mammals have a similar number of different SINEs but display lower editing levels as compared to humans (Morse et al., 2002; Waterston et al., 2002; Mattick, 2003; Kim et al., 2004a; Eisenberg et al., 2005; Mattick and Makunin, 2005; Matzke et al., 2007; Mehler, 2008; Ghildiyal and Zamore, 2009). For example, structured mRNAs or mRNAs with edited 3′ UTRs interact with P54^nrb^ complexes and are retained in the nucleus (Chen and Carmichael, 2008). In mouse, CAT2 transcribed nuclear RNA (CTN-RNA), whose 3′ UTR is edited, is localized to paraspeckles (Prasanth et al., 2005). Studies utilizing eukaryotic mutants show that A to I RNA editing is an absolute requirement for normal functioning of the nervous system (Reenan, 2001; Tonkin et al., 2002; Wang et al., 2004a). Not surprisingly, deregulation of RNA editing results in dysregulation of the nervous system (Kwak and Kawahara, 2005; Valente and Nishikura, 2005). Abnormal RNA editing has been implicated in epilepsy, schizophrenia, depression, suicide, prion-induced neurodegeneration, autosomal dominant episodic ataxia type I, Prader Willi syndrome (PWS), ALS, AD, and HD (Niswender et al., 1999; Sodhi et al., 2001; Gurevich et al., 2002; Iwamoto and Kato, 2003; Englander et al., 2005; Iwamoto et al., 2005; Kwak and Kawahara, 2005; Valente and Nishikura, 2005; Kishore and Stamm, 2006; Doe et al., 2009; Morabito et al., 2010; Singh et al., 2011; Kiesel et al., 2012).

Brain region-specific changes at the Q/R editing site of the GluR-B transcript have been described in both AD and HD (Akbarian et al., 1995a,b; Wright and Vissel, 2012). The pathology of sporadic ALS, a progressive neurodegenerative disease of the motor neurons, is due to glutamate excito-toxicity, where increased glutamate levels activate glutamate-gated ion channels that results in excessive Ca^++^ influx and neuronal death. Of note, A to I RNA editing has been implicated in excessive Ca^++^ influx in ALS. Recently, it has been shown that there is a strong correlation between increased editing in EAAT2 pre-mRNA and activation of alternative poly A site in the motor cortex of ALS patients (Flomen and Makoff, 2011). On the other hand, ALS patients also have decreased editing of the Q/R site in GluR-B transcripts of spinal motor neurons (62–100% relative to controls with 100% editing; Kwak and Kawahara, 2005; Kwak et al., 2008). Collectively, these observations suggest a differential deregulation of A to I editing in ALS likely leads to disturbances in the Ca^++^ permeability and neuronal cell death characteristic of this disease (Akbarian et al., 1995b).

Pharmacological studies provide a clear role for serotonin in psychiatric disorders such as schizophrenia, depression, and anxiety (Kennett et al., 1997; Martin et al., 1998). Editing of the 5HT_2C_R mRNA is involved in the pathophysiology of psychotic disorders. Specifically, editing regulates the efficacy of hallucinogenic and antipsychotic drugs on the 5HT_2C_R (Sodhi et al., 2005). Abnormal editing of the 5HT_2C_R is associated with hyperphagia, schizophrenia, depression, and suicide in humans (Niswender et al., 1999; Sodhi et al., 2001; Gurevich et al., 2002; Iwamoto and Kato, 2003; Morabito et al., 2010) and abnormal editing is also observed in animal models of affective disorders and PWS-like (Englander et al., 2005; Iwamoto et al., 2005; Kishore and Stamm, 2006; Doe et al., 2009; Morabito et al., 2010; Singh et al., 2011). In animals, 5HT_2C_R editing is sensitive to stress and medication as well as the genetic background and behavioral state of animals (Iwamoto et al., 2005; Du et al., 2006; Gardiner and Du, 2006; Hackler et al., 2006). These studies imply that animal models may provide additional insights into the relationship between editing of the 5HT_2C_R and clarify findings from postmortem brains of patients with mental disorders. Given to the functional implications of RNA editing of substrates implicated in these diseases, the ADAR enzymes represent novel targets to treat psychiatric disorders.

## DYSREGULATED ncRNAs IN NEURODEGENERATION

Research in the role of ncRNAs in neurodegeneration has exploded in recent years, with several in-depth review articles available (Mattick and Makunin, 2006; Mehler and Mattick, 2007; Dinger et al., 2008; Mattick and Mehler, 2008; Eacker et al., 2009; Lau and de Strooper, 2010; Sonntag, 2010; Bian and Sun, 2011; Qureshi and Mehler, 2011, 2012; Enciu et al., 2012; Junn and Mouradian, 2012; Saito and Saito, 2012). In the following sections, an overview of the impact of dysregulated ncRNAs in neurodegeneration is provided, with a focus on the role of RNA editing in the pathophysiology. To date, RNA editing has only been documented in miRNAs, but it is highly plausible that RNA editing occurs in other ncRNA species. Thus, also included is a description of key research findings in the role of other ncRNAs, such as snoRNAs and lnRNAs, in neurodegenerative diseases. These ncRNA species are ripe for research to analyze whether RNA editing might impact their expression and activity. Furthermore, the fact that binding of ADARs in the absence of editing also affects miRNA biogenesis supports the hypothesis that ADARs can interfere with the function of other ncRNAs.

## DYSREGULATED miRNAs IN NEURODEGENERATION

The biological significance of edited ncRNAs remains unknown. However, the possibility of a role in RNA interference is likely (Haramati et al., 2010; Hansen et al., 2012). RNA editing regulates precursor miRNAs that play a role in the biogenesis and function of certain miRNAs, which are abundantly edited by ADARs (Alon et al., 2012; Vesely et al., 2012). Several pri-miRNA are A to I edited, which can prevent processing at either stages or modify the targets of the final RISC complex (Kawahara et al., 2007). The fact that neural defects including tremors and neurodegeneration are present in ADAR-knockout *Drosophila melanogaster* makes a strong case for requirement of regulated A to I editing for normal behavior and nervous system functioning (Palladino et al., 2000; Keegan et al., 2005; Jepson and Reenan, 2010).

Non-coding RNAs, such as miRNAs, siRNAs, and piRNAs all guide effector Argonaute protein to either genomic loci or target RNAs in a sequence-specific manner (Mattick, 2003; Mattick and Makunin, 2005; Pang et al., 2006). Development and neural cell differentiation are regulated by brain-specific miRNAs (Haramati et al., 2010; Hansen et al., 2012). In the adult brain at various time points, miRNAs are known to regulate neural function and synaptic plasticity. Expression of miRNAs are tightly regulated during developmental processes, cell proliferation, neuronal gene expression, brain morphogenesis, neural cell fate, apoptosis, and stem cell division (Mattick, 2003; Mattick and Makunin, 2005; Matzke et al., 2007; Mehler, 2008; Ghildiyal and Zamore, 2009). The brain displays both temporal and region-specific miRNA expression, and the most abundant miRNA expression is observed in cerebellum and cerebral cortex (Haramati et al., 2010; Hansen et al., 2012). Depending on where miRNAs are localized, distinct miRNAs are also involved in memory formation (Hansen et al., 2012).

MicroRNAs can be derived from either the introns or exons of both protein-coding and ncRNAs transcribed by RNA polymerase II. Processed small hairpin RNAs or double-stranded RNA precursors give arise to miRNAs that are generally 22nt long (Bartel, 2004). miRNAs that contain 21–23 nucleotide regulatory sequences inhibit translation of targeted mRNA by base pairing with the targeted regions in the mRNA (Pal-Bhadra et al., 2002; Kuersten and Goodwin, 2003; Nishikura, 2006; Carthew and Sontheimer, 2009). A number of miRNAs are associated with neuropsychiatric diseases via silencing of targets involved in disease development. Perturbations of miRNA expression, sequence, or target sites are all associated with numerous neuronal diseases. During neuroblast differentiation, double-stranded RNA silencing element exclusive to neuronal cells, i.e., mir-124a, dictates transcriptional activation of a silencing factor (NRSF; Chen et al., 2012). Increased expression of mir-21 is linked to glioblastoma (Wang et al., 2012). Deregulated DGCR8 expression, which is associated with DiGeorge syndrome (Chen et al., 2012), is involved in miRNA processing and in learning disability. Tourette’s syndrome is associated with sequence variations in mir-189, which targets SLIT (axonal growth-controlling protein SLIT) in a mechanism that includes alterations in the miR-189 binding site in SLIT and Trk-like family member1 (SLITRK-1) mRNA (Abelson et al., 2005), a protein that is essential for neuronal growth, guidance, and neurite branching. Deregulated mir-175 expression has been linked to the X-linked mental retardation (MRX3), which resembles an early onset PD. In Waisman syndrome, disruption of the 3′ UTR of fibroblast growth factor 20 (FGF20) by a mutation alters the recognition site of mir-433, which results in increased translation of FGF20 and is correlated with increased alpha-synuclein expression (Dostie et al., 2003; Wang et al., 2008; Harraz et al., 2011). All together these studies suggest that alterations in miRNA expression lead to dysregulated neuronal functioning.

## DYSREGULATED SMALL NUCLEOLAR RNAs IN NEURODEGENERATION

While a role for ADARs in other ncRNAs has not been reported, ncRNAs form secondary structures that might be recognized by ADARs. Thus, it is possible that RNA editing or ADAR binding might alter structure and function of other ncRNAs described in the following sections. SnoRNAs range from 60 to 300 nucleotides in length and guide site-specific modification of nucleotides in target RNAs by base pairing with short regions of target RNA. SnoRNAs can be divided into two major classes: the box C\D snoRNAs, which guide 2′-O-ribose-methylation, and H/ACA snoRNAs that guide pseudouridylation of target RNAs (Kiss et al., 2002, 2004). Another group known as orphan snoRNAs because of their unknown RNA targets have been identified. Targets include ribosomal RNA (rRNA), small nuclear (snRNAs), and mRNAs (Pang et al., 2006). Mammalian snoRNAs are derived from introns of coding or non-coding genes (Meier, 2005). C\D snoRNAs are localized to nucleolus, whereas H/ACA snoRNAs are localized to cajal bodies. snoRNAs exhibit tissue-specific, developmental, and imprinting-regulated expression (Rogelj and Giese, 2004).

A number of brain-specific snoRNAs have been identified in mice including MBI-36, MBII-13, MBII-48, MBII-49, MBII-52, MBII-78, and MBII-85 (Ding et al., 2005). These snoRNAs display brain region-specific expression and play crucial roles in gene regulation and normal physiology. Similarly homolog of these snoRNAs is also highly enriched in the human brain. Some of these snoRNAs have been shown to be associated with contextual memory consolidation (fear conditioning; Rogelj et al., 2003). Interestingly, snoRNA MBII-52 targets the serotonin 2C receptor (5HT_2C_R) and regulates alternative splicing and editing of the 5HT_2C_R (Kishore and Stamm, 2006). As described above, editing of the 5HT_2C_R has been implicated in depression, anxiety, schizophrenia, and feeding regulation (Niswender et al., 1999; Sodhi et al., 2001; Gurevich et al., 2002; Iwamoto and Kato, 2003; Hackler et al., 2006; Singh et al., 2007, 2009, 2011). Abnormal MBII-52 and MBII-85 snoRNA expression (Ding et al., 2005; Kishore and Stamm, 2006; Kishore et al., 2010) and A to I editing of the 5HT_2C_R editing are all implicated in PWS and obesity (Kishore and Stamm, 2006; Kishore et al., 2010; Morabito et al., 2010; Schellekens et al., 2012). Of note, ADAR2 is sequestered in the nucleolus (Desterro et al., 2003; Sansam et al., 2003), and ADAR2-mediated editing of RNA substrates in the nucleolus is inhibited by snoRNAs (Vitali et al., 2005). Taken together, these multiple studies suggest that a very complex RNA regulatory network maintains homeostasis in the CNS (Kishore and Stamm, 2006; Kishore et al., 2010). It is unclear how editing or splicing of the 5HT_2C_R contributes to pathogenesis of PWS. However, the snoRNA SNORD-52 involvement of RNA editing and splicing of the 5HT_2C_R and the abnormal pattern of expression of the 5HT_2C_R or altered edited 5HT_2C_R ratio in PWS suggests that a compromised 5-HT signaling might contribute to pathogenesis of the disease (Cavaille et al., 2000, 2002; Kishore and Stamm, 2006; Kishore et al., 2010). This idea is supported by data in genetically modified mice expressing only the fully edited 5HT_2C_R, which mimics a PWS-like phenotype (Morabito et al., 2010). In addition, many other snoRNAs are mapped to the Prader Willi locus such as HBII-13, HBII-52, and HBII-85, and these may also be involved in or be regulated by imprinting (Rogelj and Giese, 2004).

## DYSREGULATED LONG NON-CODING RNAs IN NEURODEGENERATION

Numerous brain-specific lncRNAs are alternatively spliced, developmentally regulated, and are physiologically responsive (Furuno et al., 2006; Kapranov et al., 2007). lncRNAs that are derived from the mammalian genome are both polyadenylated and non-polyadenylated (Mattick and Gagen, 2001; Huang et al., 2012; Song et al., 2012). Imprinting and antisense transcription of lncRNAs that host genes for miRNAs and snoRNAs that are localized to the nucleus of nervous tissue suggest that these lncRNAs may be involved in gene regulation (Furuno et al., 2006). The transcription patterns of lncRNAs are in complex intergenic, overlapping, and antisense patterns relative to adjacent protein-coding genes (Satoh, 2012; Tran et al., 2012). Thus, it is possible that the lncRNAs regulate the expression of those genes. LncRNAs are involved in the formation and function of cellular organelles that are regulated transcriptionally and developmentally in a cell-specific manner (Satoh, 2012; Tran et al., 2012). The functions of numerous lncRNAs are not known but studies suggest that they play an important role in cell identifying the neuronal and glial cells in the CNS (Mercer et al., 2010).

Patients with HD have widespread changes in their brain gene regulatory networks (Ideker and Sharan, 2008). These changes include non-protein coding RNAs and protein coding RNAs. Seven lncRNAs in the human brain are specifically dysregulated in HD (Johnson, 2012). New findings suggest that, besides protein-coding genes, ncRNAs also contribute to neurodegenerative processes. Evidence for a role for ncRNAs in HD comes from the genome-wide data where novel, non-coding targets of RE1-silencing transcription factor (REST) were discovered (Buckley et al., 2010; Johnson et al., 2010). A human accelerated region 1 (HAR1) specifically is transcribed in the nervous system. REST is targeted to the HAR1 locus that is recognized by specific DNA regulatory motifs and results in potent transcriptional repression. Aberrant nuclear localization of the master transcriptional repressor REST disrupts the gene regulatory networks in the neurons of HD patients. Notably, HAR1 levels are significantly lower in the striatum of HD patients compared with unaffected individuals. Interestingly, many of these lncRNAs contain genomic binding sites for the transcriptional repressor REST, a key mediator of transcriptional changes in HD, including the known REST target lncRNA, DGCR5. LncRNAs TUG1 (necessary for retinal development), and NEAT1 (a structural component of nuclear paraspeckles) are upregulated in HD caudate, while the brain-specific tumor-suppressor MEG3 is downregulated in HD (Johnson, 2012). Formation of epigenetic ribonucleoprotein complexes, including lncRNAs TUG1 and MEG3, regulates gene expression. All together these findings suggest that changes in lncRNA expression are widespread in HD, contributing to altered epigenetic gene regulation in diseased neurons and likely corresponding neurodegeneration. Thus, studying the regulation of non-coding gene expression changes and lncRNA network changes in HD may provide a better understanding of and suggest novel treatments for not only HD but also other neurodegenerative processes. For example, the lncRNA BACE 1 has been directly implicated in upregulation of amyloid-beta 1–42 in AD (Faghihi et al., 2008). Thus lncRNAs play an important role in regulating gene expression for normal functioning of the nervous system.

## DYSREGULATED IMPRINTED NON-CODING RNAs IN NEURODEGENERATION

Imprinted genes are known to play essential roles in both neural development and adult CNS functioning. Alterations in their expression profiles are linked to a spectrum of complex neurodevelopment and neuropsychiatric disorders (Costa, 2005; Davis et al., 2005). These allele-selective genes exhibit preferential and exquisite cell-specific patterns of expression within the brain and are frequently processed from larger transcriptional units that encompasses multiple tandem repeats of snoRNAs and miRNAs (Sleutels et al., 2000; Costa, 2005; Davis et al., 2005; Lewis and Reik, 2006). These imprinted loci usually generate a complex spectrum of spliced and unspliced larger ncRNAs of unknown function (Sleutels et al., 2000; Costa, 2005; Davis et al., 2005; O’Neill, 2005; Furuno et al., 2006). Additional ncRNAs are associated with imprinted loci that include the production of antisense RNAs to reciprocally imprinted neighboring protein-coding genes (Sleutels et al., 2000; Davies et al., 2005). The role of imprinted genes in regulating distinct brain signaling systems and in mediating brain–behavior relationships can be deduced from spectrum of neurological diseases caused by disruptions in imprinted loci: PWS and Angelman syndromes, autism, schizophrenia, attention deficit hyperactivity disorder, bipolar disorder, and Tourette’s syndrome (Davies et al., 2004, 2005, 2006; Wang et al., 2004b).

## DYSREGULATED TRANSFER AND RIBOSOMAL RNAs IN NEURODEGENERATIVE DISEASE

Adenosine deaminase that act on RNA have the ability to act in concert with ADATs to modify transfer RNAs (tRNAs) to change codon recognition. Interestingly, a mutation in the “editing” domain of a specific aminoacyl-tRNA synthetase results in mischarged tRNAs, intracellular accumulation of misfolded proteins in neurons, and induction of the endoplasmic reticulum-mediated unfolded protein stress response with associated neurodegeneration (Lee et al., 2006). tRNAs and rRNAs are implicated in a broad array of neural developmental and mature CNS functions. Not surprisingly, therefore, mutations in these two classes of ncRNAs underlie a range of neurodevelopment, neurodegenerative, and neuropsychiatric diseases. Such examples include chronic progressive external ophthalmoplegia (CPEO), Kearn–Sayre syndrome (KSS: CPEO with retinal degeneration), mitochondrial myopathy, encephalopathy, lactic acidosis, and stroke (MELAS) syndrome that manifests in mitochondrial encephalopathy with stroke-like syndromes and migraine headaches, myoclonic epilepsy with ragged red fibers (MERRF) syndrome that results in myoclonus epilepsy, mitochondrial myopathy, cerebella ataxia (Dimauro, 2004; Dimauro and Davidzon, 2005; Fattal et al., 2006), and motor neuron disease (Borthwick et al., 2006). Other tRNA-mediated neuropsychiatric diseases include schizophrenia, psychosis, delirium, personality disorders, major depressive disorders, and anxiety disorders (Fattal et al., 2006). Besides tRNA-associated diseases, deregulated rRNA is also implicated in RNA oxidation of vulnerable neurons in AD (Honda et al., 2005).

## DYSREGULATED RNA TRINUCLEOTIDE EXPANSIONS IN NEURODEGENERATION

The expansion of trinucleotide repeats caused by RNA-mediated mechanisms is associated with neurodegenerative diseases (Gallo et al., 2005; Gatchel and Zoghbi, 2005). Dramatically expanded (>200) CGG repeats in the 5′ UTR of the Fmr1 gene results in fragile X syndrome. The related disease is also associated with smaller (60–200) trinucleotide repeat expansion called fragile X tremor/ataxia syndrome (FXTAS). FXTAS is associated with tremor, cerebella ataxia, cognitive decline, peripheral neuropathy, PD, autonomic dysfunction, proximal muscle weakness, multisystem atrophy, and dementia (Hagerman et al., 2005; Van Esch, 2006). Myotonic dystrophy, another trinucleotide disorder, is predominantly a muscle disorder which exists in two neurological forms: DM1 with mental retardation, memory, visuo-spatial, and executive dysfunction, and DM2 with preferential executive dysfunction (D’Angelo and Bresolin, 2006). DM1 is associated with CTG expansion within the 3′ UTR of the dystrophia myotonica protein kinase (DMPK) gene, and DM2 is linked to CCTG expansion in intron 1 of the zinc finger protein gene ZNF9 (Brook et al., 1992; Fu et al., 1992; Mahadevan et al., 1992; Ranum et al., 1998). These mutant RNAs orchestrate different forms of pathogenesis depending on the degree and type of expanded repeat length and their molecular interactions with the muscleblind-like (MBNL) family of RNA-binding proteins (Jiang et al., 2004; Pascual et al., 2006).

Several forms of spinocerebellar ataxia (SCA) are also implicated in different RNA-mediated pathological mechanisms. SCA8 results from CTG expansion of the 3′ UTR of an untranslated antisense RNA with partial overlap with the Kelch-like 1 (KLHL1) gene (Koob et al., 1999; Nemes et al., 2000; Mutsuddi et al., 2004; Gatchel and Zoghbi, 2005). Utilizing SCA8 as a modifier screen, four novel ncRNAs have been identified that show preferential neuronal expression (Mutsuddi et al., 2004). SCA10 is mediated by an unstable ATTCT repeat expansion in the 3′ end of a large intron of a gene of unknown function that may result in transcriptional silencing or in a different RNA-associated toxic mechanism (Matsuura et al., 2000). SCA12 is caused by CAG expansion in the non-coding 5′ promoter or 5′ UTR of the PPP2R2B gene, which encodes a brain-specific regulatory subunit of protein phosphatase 2A (Holmes et al., 1999). All together these findings suggest that, depending on where the expanded trinucleotide repeat is localized, disease pathogenesis is likely mediated by distinct trans-dominant RNA or alternatively by toxic gain of function mechanisms (Holmes et al., 2003). Taken together, these studies suggest that elucidating the lncRNA network is an important step toward understanding neurodegeneration and may reveal new targets to treat neurodegenerative diseases.

## SUMMARY

RNA is a carrier of information and plays a central role in regulating development. A variety of regulatory non-protein-coding RNA molecules form complex multi-layered biological systems. A gene regulatory network that allows normal functioning of the CNS governs this complex system. A deregulated complex gene regulatory network plays a significant role in common neurodegenerative diseases. Furthermore, the list of known ncRNAs implicated in mammalian brain health and disease is growing. RNA can alter the information in the genetic code without altering the hard-wired DNA through splicing and RNA editing. ADAR substrates involved in RNA editing mechanisms provide functional complexity. RNA editing mediates the environmental cues by transmitting information to the epigenome. This mechanism connects the environment to the genome and plays important roles in a broad range of processes, from evolution to learning and memory. A to I RNA editing, besides altering protein function, also has the potential to alter splice site choice, miRNA target diversity, miRNA processing, and perhaps chromatin architecture. Furthermore, RNA editing alters RNA structure and thereby could potentially impact the biological functions of multiple types of ncRNAs. Therefore, RNA editing, RNA modification, small and long ncRNAs, and their complex regulatory network lead to a unifying theme of RNA-mediated regulatory circuitry for normal brain function.

## Conflict of Interest Statement

The author declares that the research was conducted in the absence of any commercial or financial relationships that could be construed as a potential conflict of interest.
